# Ferrous Citrate Up-Regulates the NOS2 through Nuclear Translocation of NFκB Induced by Free Radicals Generation in Mouse Cerebral Endothelial Cells

**DOI:** 10.1371/journal.pone.0046239

**Published:** 2012-09-28

**Authors:** Li-Ching Chen, Chin Hsu, Chuang Chin Chiueh, Wen-Sen Lee

**Affiliations:** 1 Graduate Institute of Medical Sciences, School of Medicine, Taipei Medical University, Taipei, Taiwan; 2 Department of Physiology, Kaohsiung Medical University, Kaohsiung, Taiwan; 3 Taipei Medical University-Shuang Ho Hospital, Taipei, Taiwan; 4 Department of Physiology, School of Medicine, Taipei Medical University, Taipei, Taiwan; 5 Cancer Research Center, Taipei Medical University Hospital, Taipei, Taiwan; Kaohsiung Chang Gung Memorial Hospital, Taiwan

## Abstract

Previous studies indicate that the inducible nitric oxide synthase 2 (NOS2) of the brain vascular tissue in experimental subarachnoid hemorrhage (SAH) rats is a critical factor for inducing cerebral vasospasm. However, the underlying molecular mechanisms remain to be elucidated. Here, we applied ferrous citrate (FC) complexes to the primary cultured mouse cerebral endothelial cell (CEC) to mimic the SAH conditions and to address the issue how SAH-induced NOS2 up-regulation. Using immunocytochemical staining technique, we demonstrated that NOS2 was expressed in the cultured CEC. Treatment of the CEC with FC induced increases of the intracellular level of ROS, nuclear factor kappa-light-chain-enhancer of activated B cells (NFκB) nuclear translocation as well as NFκB binding onto the NOS promoter, and the levels of NOS2 mRNA and protein. These effects were abolished by pre-treatment of the cell with N-Acetyl-Cysteine (NAC), a reactive oxygen species (ROS) scavenger. In the present study, two previously predicted NFκB binding sites were confirmed in the NOS2 promoter within the range of −1529 bp to −1516 bp and −1224 bp to −1210 bp. Interestingly, both NFκB binding sites are involved in the FC-activated NOS2 transcriptional activity; the binding site located at −1529 bp to −1516 bp played a greater role than the other binding site located at −1224 bp to −1210 bp in the mouse CEC. These findings highlight the molecular mechanism underlying FC-induced up-regulation of NOS2 in the mouse CEC.

## Introduction

Hemorrhage stroke, which includes intracerebral hemorrhage and SAH, is associated with high risk of mortality and morbidity. Although the hemorrhage stroke is treated, patients still face the threat of cerebral complications such as rebleeding, recurrent stroke, liquefaction, vasospasm, and hydrocephalus [1].

The pathogenesis of cerebral complications after hemorrhage stroke is complicated and still not fully understood. However, accumulating evidence has suggested that impaired iron metabolism is an initial cause of neurodegeneration, and several common neurodegenerative disorders have been proposed to be associated with dysregulation in CNS iron homeostasis [2]–[4] and small molecular weight iron complexes [5]. Iron functions as an important cofactor in cellular energy production and contributes to the activity of many proteins and mitochondrial enzymes in most living tissues [6]. Normally, iron is bound and inactivated by transport proteins (e.g. transferrin) and intracellular storage proteins (e.g. ferritin). However, the unbound iron can be found in the brain under some pathological circumstances such as intracerebral hemorrhage. The heme from red blood cells is cleaved into biliverdin by heme oxygenase in astrocytes and microglia, thereby releasing iron [7], [8]. The iron released from heme is highly toxic to neurons. Moreover, most of the non-heme iron in the brain is bound to ferritin as ferric ion, and can be released only after being reduced to the ferrous state. Reduction and release of iron from ferritin can be accomplished by superoxide, acidic pH, ascorbate and catecholamines [9], [10], which are rich in the extracellular fluid of the brain, especially during hypoxia/ischemia conditions. It has been shown that hypoxia/ischemia conditions cause neuronal cell death and the affected area is accompanied by increased brain levels of iron and ferritin in the cerebral cortex and the hippocampus [11]–[13]. It has been hypothesized that iron in the ferrous state causes vasospasm. As iron is unbound in the presence of oxygen, it catalyzes the generation of toxic hydroxyl radicals, which could contribute to SAH pathology [7]. The notion that iron plays an important role in the development of SAH was supported by intracerebroventricular injection with ferrous ammonium citrate causing increases of the level of toxic lipid peroxidation products, such as 4-hydroxynonenal (HNE), in the field CA3 of the hippocampus in a rat model [14], and intravenous administration with 2,2′-dipyridyl, an iron chelator, prevents delayed vasospasm in a primate model of SAH [15]. Furthermore, desferal chelates iron complex and prevents the iron-catalyzed oxidative stress and brain injury *in vivo* [16].

Nitric oxide synthase (NOS) consist of different subtypes depending on the tissue type including neuronal (NOS1), inducible or macrophage (NOS2), and endothelial (NOS3) enzyme [17]. NOS1, previously known as non-inducible nNOS, can be induced by ROS generated by serum deprivation or preconditioning stress leading to induction of the redox protein thioredoxin, manganese superoxide dismutase (MnSOD), or Bcl-2 for cytoprotection and survival. NOS2 is inducible and previously named as iNOS. Moreover, NOS3 can also be induced in endothelial cells and lead to vasodilation exerting a protective effect in the early stages of ischemia. ROS can regulate all three subtypes of NOS expression [3], [18]–[20].

Previous *in vivo* studies inferred that an increase of NOS2 expression might play a critical role in the occurrence and progression of the SAH-induced vasospasm [21], [22]. However, the molecular mechanisms underlying SAH-induced NOS2 up-regulation is still unclear. In the present study, we applied FC complexes to the mouse primary cultured CEC to mimic the SAH conditions and to address the issue how SAH-induced NOS2 up-regulation.

**Figure 1:**
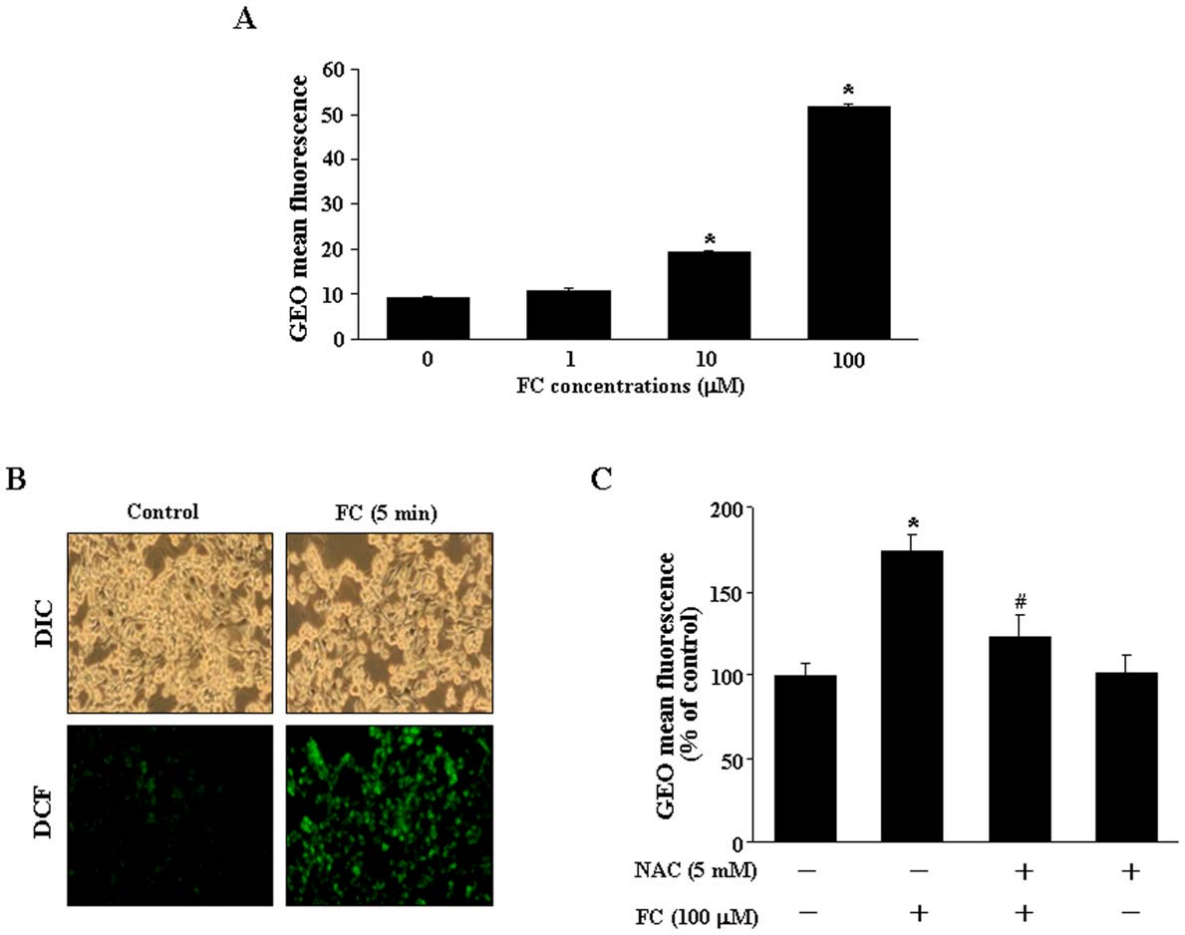
FC increases ROS generation in the CEC. (A) FC (1–100 µM) concentration-dependently increased ROS generation in the CEC. (B) The ROS generation was observed at 5 min after FC (100 µM) treatment. (C) FC (100 µM)-induced increases of ROS generation in CEC were prevented by pretreatment of the cell with a ROS scavenger, NAC (5 mM). ROS levels were assayed using 5 µM DCF as described in Materials and Methods. DCF fluorescence images and DIC images were taken using Leica TCS SP5 fluorescent microscope imaging system (Wetzlar, Germany) and the levels of ROS were quantified by flow cytometric analysis. Values represent the means±s.e.mean. (n = 4). ^*^ *P* < 0.05 different from control. ^#^ *P* < 0.05 different from FC-treated group. DIC, differential interference contrast; DCF, dichlorodihydrofluorescein diacetate [CM-H2DCF-DA].

## Materials and Methods

### Chemicals

N-acetylcysteine (NAC) was purchased from Sigma-Aldrich (St. Louis, MO, USA). Bay 11-7082, a selective IkappaB kinase (IKK) inhibitor was obtained from Cayman Chemical (Ann Arbor, MI). PDTC, an NFκB inhibitor, was purchased from Sigma-Aldrich. Chemicals used in this study were dissolved in dimethyl sulfoxide (DMSO) or water according to the manufacturer’s protocol.

**Figure 2:**
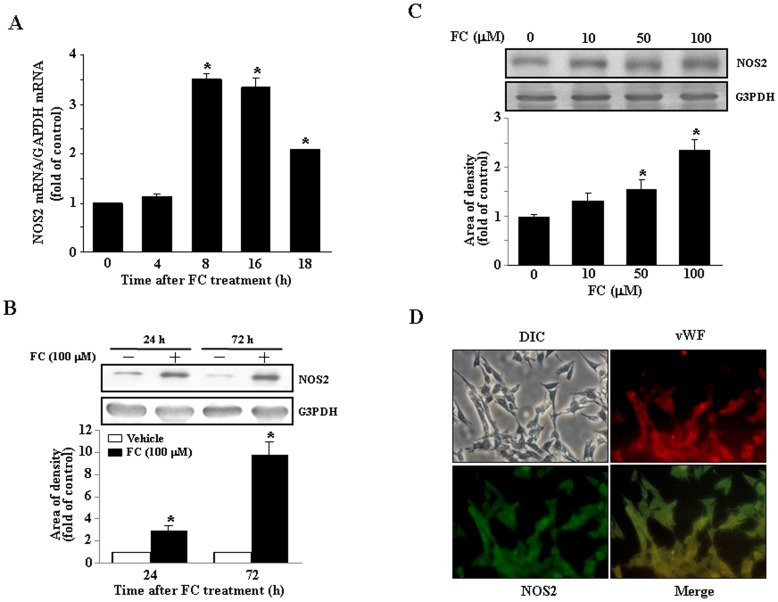
Effects of FC on NOS2 expression in the CEC. (A) The levels of NOS2 mRNA in the CEC were significantly increased at 8 h after FC treatment. A peak of NOS2 mRNA level was observed at 8–16 h after FC treatment, and then began to decline. The levels of NOS2 mRNA were determined using quantitative real time-PCR, adjusted with G3PDH mRNA, and expressed as ratio over control. (B) FC (100 µM) treatment increased the levels of NOS2 protein in the CEC. FC (100 µM) time-dependently increased the level of NOS2 protein. Top panel: representative results of NOS2 and G3PDH protein levels determined by Western blot analysis. Bottom panel: quantitative results of NOS2 protein levels, which were adjusted with G3PDH protein level and expressed as fold-induction of its own control. Values represent the means±s.e.mean. (n = 3). ^*^ *P* < 0.05 different from corresponding control. (C) The FC-induced increases of the level of NOS2 protein were in a concentration-dependent manner. Top panel: representative results of the levels of NOS2 and G3PDH protein determined by Western blot analysis. Bottom panel: quantitative results of NOS2 protein levels, which were adjusted with G3PDH protein level and expressed as fold of control. Values represent the means±s.e.mean. (n = 3). ^*^ *P* < 0.05 different from corresponding control. (D) FC-induced NOS2 expression is mainly located in the CEC. Micrographs show NOS2 (green) and vWF (red) immunoreactivity detected by dual immunofluorescent staining as described in Materials and Methods. NOS2, inducible nitric oxide synthase 2; G3PDH, glyceraldehyde3-phosphate dehydrogenase, vWF, von Willebrand Factor.

### Cell Culture

The CEC was prepared as previously described [23] and all procedures were performed according to the Taipei medical university animal care and use rules (licenses No. LAC-97-0160) and an Association for Assessment and Accreditation of Laboratory Animal Care approved protocol. The surgery was performed under isoflurane anesthesia to minimize suffering. Briefly, the Balb/c mouse was sacrificed by decapitation, meninges and white matter were removed, and cortices were minced and gently dissociated in Hank’s balanced salt solution (GIBCO, Grand Island, NY). The resulting microvessel fraction was then sequentially digested with collagenase/dispase at a concentration of 1 mg/mL (Sigma-Aldrich, St. Louis, MO) for 6 h at room temperature. After centrifugation, the pellet containing the CEC was washed with Dulbecco’s modification of Eagle’s medium (DMEM, GIBCO), maintained in DMEM supplemented with 10 % fetal bovine serum (FBS) in a humidified incubator (37°C, 5% CO_2_). CEC showed positive immunoreactivity for Von Willebrand factor (vWF), a marker for endothelial cells. Cells from passages 10 to 25 were used.

**Figure 3:**
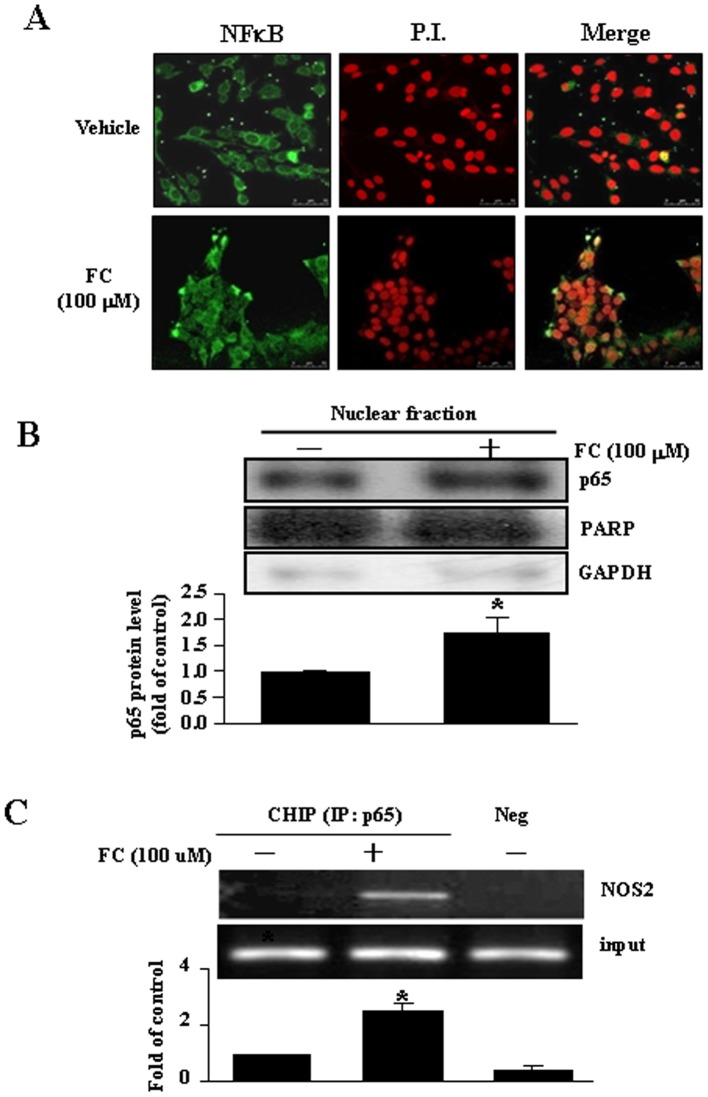
Involvement of NFκB (p65) in regulating the NOS2 promoter activity in the FC-treated CEC. (A) FC (100 µM) induced NFκB translocation from the cytosolic fraction into the nuclear fraction in the CEC. After treatment with FC for 4 h, the CEC was fixed and then labeled with an anti-NFκB antibody, followed by an FITC-conjugated secondary antibody. The nuclei were visualized with propidium iodide (50 µg/mL) staining as described in Materials and Methods. (B) FC (100 µM) increased nuclear translocation of NFκB in the CEC. Top panel: representative results of NFκB, PARP and G3PDH protein levels determined by Western blot analysis. Bottom panel: quantitative results of NFκB protein levels, which were adjusted with PARP protein level and expressed as fold of control. Values represent the means±s.e.mean. (n = 4). ^*^ *P* < 0.05 different from corresponding control. (C) FC (100 µM) induced an increase of NFκB binding onto the NOS2 promoter. The levels of NFκB binding onto the NOS2 promoter were assessed by using ChIP assay (top panel) and quantitative real-time PCR (bottom panel). Values represent the means±s.e.mean. (n = 4). ^*^ *P* < 0.05 different from control. P.I., propidium iodide.

### FC Treatment

The FC complex was prepared as previously described [14], [24]. Ferrous ammonium sulfate and citric acid (Sigma-Aldrich) were dissolved in sterile distilled water and the pH was adjusted to 7.4 with NaOH. A concentration of 100 µM FC was freshly prepared prior to each treatment by mixing 1∶1 volume of 200 µM ferrous ammonium sulfate and 200 µM citric acid.

**Figure 4:**
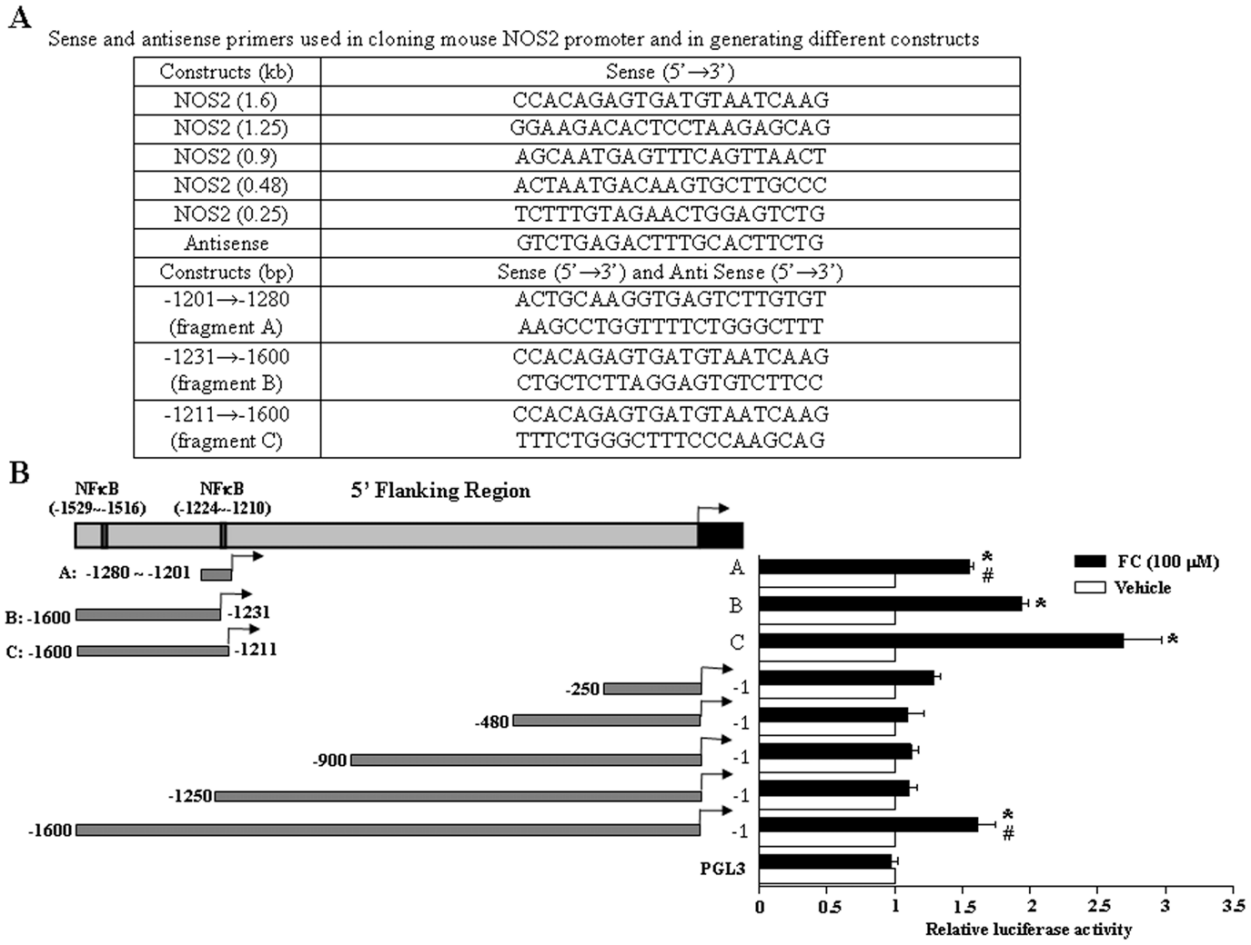
NFκB-binding sites are required for the FC-induced NOS2 promoter transactivation. 5′-serial deletions of the mouse NOS2 1.6-kb promoter were generated by PCR as described in “Materials and Methods” by using the primers indicated in (A). (B) The CEC was transiently transfected with various constructs for 24 h, and then treated with vehicle or FC (100 µM) for 16 h. Subsequently, the cell was processed for the luciferase activity assay. Quantitative results of the NOS2 promoter activity were shown and expressed as fold induction of the CEC transfected with PGL3 (control). Values represent the means±s.e.mean. (n = 4). ^*^ *P* < 0.05, different from cells transfected with PGL3; ^#^ *P* < 0.05, different from cells transfected with −1211 to −1600. C, Control.

### Measurement of ROS

For measurement of intracellular ROS levels, cells were incubated with 5 µM 5- (and-6)-chloromethyl-2,7-dichlorodihydrofluorescein diacetate acetyl ester [CM-H2DCFDA (here referred to as DCF) (Invitrogen, Carlsbad, CA)] for 10 min, and then washed with phosphate-buffered saline (PBS). The fluorescence and differential interference contrast images were taken using Leica TCS SP5 fluorescent microscope imaging system (Wetzlar, Germany) and analyzed by flow cytometry according to the manufacturer’s instructions (Becton Dickinson, San Jose, CA).

**Figure 5:**
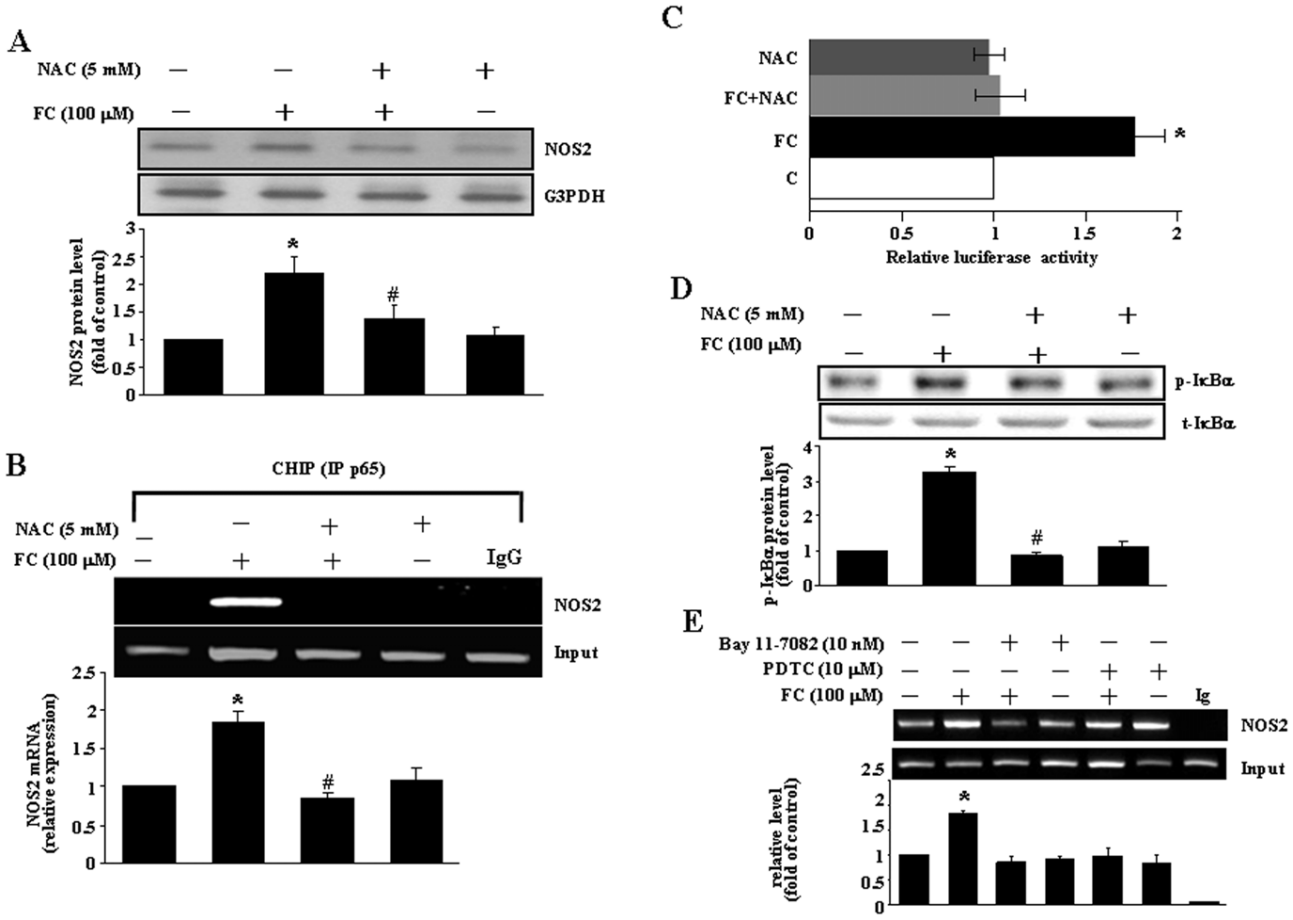
FC induces NFκB activation through increases of the levels of ROS and phosphorylated IκBα. (A) Pretreatment of the CEC with a ROS scavenger, NAC (5 mM), for 1 h prevented the FC-induced increases of the levels of NOS2 protein. Top panel: representative results of NOS2 and G3PDH protein levels determined by Western blot analysis. Bottom panel: quantitative results of NOS2 protein levels, which were adjusted with G3PDH protein level and expressed as fold of control. Values represent the means±s.e.mean. (n = 3). ^*^ *P* < 0.05 different from corresponding control; ^#^ *P* < 0.05 different from the FC-treated CEC. (B) The FC (100 µM)-induced an increase of the NFκB (p65) DNA binding onto the NOS2 promoter was completely abolished by pretreatment of the cell with NAC (5 mM). The NOS2 DNA binding activity was assessed by using ChIP assay and detected by semiquantitative PCR (top panel) and quantitative real-time PCR (bottom panel). Values represent the means±s.e.mean. (n = 3). ^*^ *P* < 0.05 different from corresponding control; ^#^ *P* < 0.05 different from the FC-treated CEC. (C) FC-induced an increase of the NOS2 promoter activity was abolished by pretreatment of the cell with NAC. Values represent the means±s.e.mean. (n = 3). ^*^ *P* < 0.05, different from control. (D) Pretreatment of the CEC with NAC (5 mM) for 1 h prevented the FC-induced increases of the levels of phosphorylated IκBα (p-IκBα) protein. Top panel: representative results of the levels of p-IκBα and total IκBα (t-IκBα) protein determined by Western blot analysis. Bottom panel: quantitative results of p-IκBα protein levels, which were adjusted with t-IκBα protein level and expressed as fold of control. Values represent the means±s.e.mean. (n = 3). ^*^ *P* < 0.05 different from corresponding control; ^#^ *P* <0.05 different from the FC-treated CEC. (E) FC increased phosphorylation of the protein IκBα and NFκB binding on the NOS2 promoter, and these effects were blocked by a ROS scavenger (NAC), an IKK inhibitor (Bay 11-7082), or NFκB translocation inhibitors (PTDC). The levels of NOS2 protein were quantified by Western blot analysis. The NFκB DNA binding activity was assessed by using ChIP assay and quantitative real-time PCR (bottom panel). Values represent the means±s.e.mean. (n = 3). ^*^ *P* < 0.05, different from corresponding control; ^#^ *P* < 0.05, different from FC-treated without ROS scavenger (NAC).

### RNA Isolation and Real-time Quantitative PCR

Total RNA was isolated from the CEC using Trizol (Invitrogen) according to the manufacturer’s protocol. The NOS2 subunit-specific primers were synthesized as described previously [25]. A LightCycler thermocycler (Roche Molecular Biochemicals, Mannheim, Germany) was used for the real-time PCR. The NOS2 mRNA fluorescence intensity was measured and normalized with the level of glyceraldehydes-3-phosphate dehydrogenase (G3PDH) using the built-in Roche LightCycler Software Version 4.

**Figure 6:**
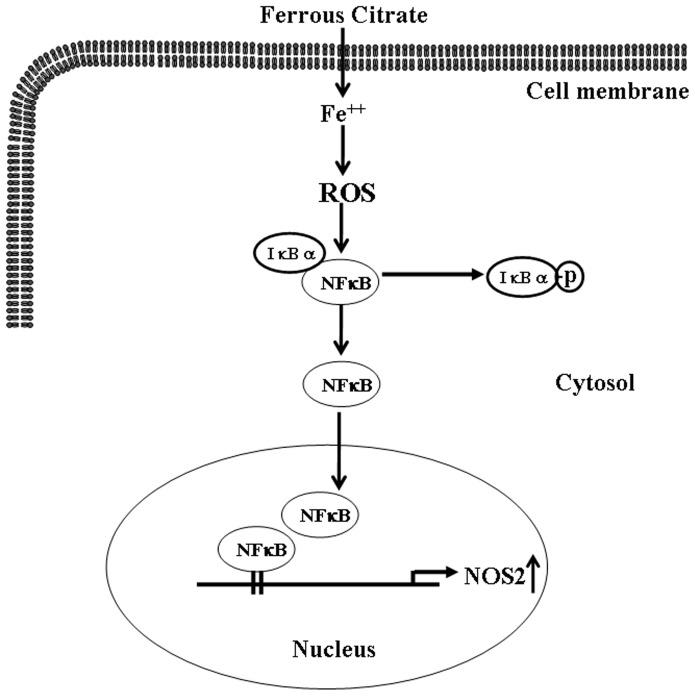
Proposed signaling pathway associated with FC-induced up-regulation of NOS2 in the CEC. FC increased ROS generation, which in turn caused IκBα phosphorylation, subsequently increased NFκB (p65) nuclear translocation and binding onto the NOS2 promoter, and finally increased the levels of NOS2 mRNA and protein.

### Reverse Transcriptase-polymerase Chain Reaction (RT-PCR) Analysis

The RT-PCR assays for NOS2 gene expression were performed as described previously [26]. The cell was treated with the FC complex for 18 h, and then processed for total RNAs isolation using Trizol reagent according to manufacturer’s protocol (Invitrogen). The cDNA was amplified from 1 µg of total RNA using a SuperScript one-step RT-PCR with platinum Taq system (Life Technologies, Karlsruhe, Germany). PCR was conducted for 35 cycles in thermal controller. Primers used for amplification were as follows: NOS2 5′-TGCTGTTCTCAGCCCAACAA-3′, and reverse: 5′-GAACTCAATGGCAT GAGGCA-3′. The polymerase chain reaction primers for G3PDH were forward primer: 5′-GCATGGCCTTCCGTGTTCCTA-3′, and reverse primer: 5′-CCTTCAGTGGGCCCTCAGATG-3′. The amplification profile involved denaturation at 94°C for 1 min, primer annealing at 58°C for 30 sec, extension at 72°C for 1 min, and repeated for 35 cycles.

### Western Blot Analysis

Western blot analysis was performed as described previously [27]. Briefly, cell lysates were prepared, electrotransferred, immunoblotted with antibodies, and then visualized by incubating with the enhanced chemiluminescence system (Amersham, Buckinghamshire, England). Monoclonal mouse NFκB and total nuclear factor of kappa light polypeptide gene enhancer in B-cells inhibitor, alpha (IκBα) antibodies, as well as polyclonal rabbit Poly ADP-ribose polymerase (PARP) and vWF antibodies were purchased from Santa Cruz, (CA, USA). Monoclonal mouse NOS2 and phospho-IκBα (p-IκBα) antibodies were purchased from BD Bioscience (Clontech) and Cell Signaling Technology (Beverly, USA), respectively. The level of G3PDH (Abcam, Cambridge, MA) was detected and used as the control for equal protein loading. The intensity of each band was quantified by densitometry analysis using Image Pro Plus 4.5 software program (Media Cybernetics, Silver Spring, MD) and pixel densities were normalized to that of the loading control in Western blot analysis.

### Immunocytochemical Staining

The CEC was seeded on glass coverslips, treated with vehicle or FC (100 µM) for 4 h, washed three times with PBS, and then fixed with 4% formaldehyde in PBS for 15 min. Cells were placed in blocking solution (PBS containing 15% FBS, 2% bovine serum albumin (BSA), and 0.1% saponin) for 45 min at room temperature, and then incubated with anti-NFκB Fluorescein isothiocyanate (FITC)-conjugated monoclonal antibody at a 1∶100 dilution for 1 h at room temperature in blocking solution. To visualize nuclei, DNA was stained with propidium iodide (50 µg/mL in PBS and 0.1% BSA) for 5 min. After washing three times with PBS, cells were viewed under a laser confocal spectral microscope imaging system (Leica, TCS SP5, Bannockburn, IL, USA).

### Chromatin Immunoprecipitation Analysis (ChIP)

ChIP assays were performed as described previously [28]. The anti-NFκB antibody (Santra Cruz) was used for immunoprecipitation reactions. Primers specific for the detection of transcription factor binding regions from −460 bp to −250 bp of the NOS2 gene were designed. The sense primer was 5′–GGATACACCACAGAGTGATG-3′, and the anti-sense primer was 5′-CATATCAGCTTCAGTCCAGC-3′.

### Construction of the *NOS2* Gene Promoter Plasmid

The 1600-bp fragment of the mouse *NOS2* promoter was amplified by genomic PCR using genomic DNA from the CEC as the template. The primers of 5′-CCACAGAGTGATGTAATCAAG-3′ and 5′ GTCTGAGACTTTGCACTTCTG-3′ were used. This PCR fragment was ligated into a basic luciferase reporter gene in the pGL3 vector (Promega, Madison, WI, USA) with Mlu1 and XhoI restriction sites, and designated as PGL3 NOS2. The pRL-TK vector encodes the Renilla luciferase gene, which was used as an internal control to normalize for pGL3 firefly luciferase expression. The sequence was confirmed by ABI 3730XL analysis system (Applied Biosystems Inc., Foster City, CA).

### Cell Transfection and Dual Luciferase Reporter Assay

For transient transfection, Lipofectamine™ 2000 transfection reagent (Invitrogen) was used according to the manufacturer’s protocol. Briefly, Lipofectamine and plasmid DNA (PGL3 NOS2 3.5 µg and 50 ng of pRL-TK) was added to each well containing cells and Opti-MEMR I Medium, and then incubated in a humidified incubator at 37°C for 4 h. The medium was replaced and the cell was then incubated for an additional 24 h. After incubation, the cell was treated with FC (100 µM) for 16 h, lysed in passive lysis buffer (Promega), and then mixed with luciferase assay substrate (Dual-Glo luciferase reporter system; Promega). The Firefly and Renilla luciferase activities were measured with a 96-well luminometer and analyzed by skanIt^TM^ software2.4.1. (Thermo Fisher Scientific, Rocford, IL).

### Statistics

All data were expressed as the mean value ± s.e.mean. Comparisons were subjected to one way analysis of variance (ANOVA) followed by Fisher’s least significant difference test. Significance was accepted at *P* < 0.05.

## Results

### FC Increases ROS Levels in the CEC

It has been indicated that iron can potentiate ROS production and exacerbate oxidative stress [29], [30]. To study the effect of iron on the production of ROS, the mouse CEC was treated with various concentrations of FC, small molecular weight iron complex, and the levels of ROS were measured by DCF. FC (1–100 µM) concentration-dependently increased the ROS level in the CEC (Fig. 1A). The FC-induced an increase of the ROS level was observed as early as 5 min after treatment (Fig. 1B). Pretreatment of the CEC with a ROS scavenger, NAC (5 mM), prevented the FC-induced increases of ROS (Fig. 1C).

### FC Increases the Levels of NOS2 mRNA and Protein in the CEC

Previously, we demonstrated that SAH caused an increase of NOS2 production in the rat basilar artery [21]. In addition, ROS has been suggested to play an important role in regulating multiple proinflammatory genes, such as *NOS1* [31] and *NOS2* [3]. Accordingly, we examined whether FC complex could induce NOS2 expression in the subcultured CEC. The level of NOS2 mRNA was significantly increased in the CEC at 8 h after FC treatment, and then began to decline at 18 h (Fig. 2A). FC (100 µM) treatment also time-dependently increased the level of NOS2 protein in the CEC (Fig. 2B). The FC-induced increases of the level of the NOS2 protein were in a concentration-dependent manner (Fig. 2C). To confirm that the expression of NOS2 protein was localized in endothelial cells but not from the culture contaminated with other cell types, immunocytochemical staining was performed. As illustrated in Figure 2D, NOS2 immunoreactivity was co-localized with Von Willebrand Factor, a marker for endothelial cells, suggesting that the NOS2 protein detected in the present study was mainly produced by the CEC. The expression of NOS3 mRNA and protein was not significantly altered (unpublished observation).

### FC Increases NFκB Nuclear Translocation and NFκB Binding onto the NOS2 Promoter

NFκB is a key transcription factor involved in the regulation of NOS2 expression [32]. The present study examined whether FC small molecular iron complexes could induce nuclear translocation of NFκB. In the control culture, NFκB was mainly located in the cytoplasm of the CEC. Following FC (100 µM) treatment, NFκB was translocated from the cytoplasm into the nucleus as evidenced by colocalization of the NFκB immunoreactivity and propidium iodide (PI) staining (Figs. 3A and 3B). FC-induced nuclear translocation of NFκB (p65) began at 30 min after treatment and lasted for 8 h (data not shown). ChIP assays revealed that the nuclear NFκB was bound to the NOS2 promoter in the CEC (Fig. 3C), suggesting that there was a transcription activation effect of FC complex on the mechanisms for NOS2 gene expression in the subcultured CEC.

### Effect of FC on NFκB-binding Sites in the Promoter Region of the NOS2 Gene

To map the regulatory regions of the mouse NOS2 promoter, the present study employed the serial deletion strategy by constructing recombinant plasmids containing different 5′-deletions of the mouse NOS2 promoter linked to a luciferase reporter gene. The full length of the mouse NOS2 5′ flanking region (1.6 kb) was cloned by PCR using mouse genomic DNA as a template (Fig. 4A). Serial deletions of the mouse NOS2 promoter (0.25 kb, 0.48 kb, 0.9 kb, and 1.25 kb) were generated by using the cloned mouse NOS2 promoter as a template and the primers were shown in Figure 4A. As shown in Figure 4B, the NOS2 promoter luciferase activity was 1.7 fold increased in the CEC transfected with the full length of the promoter (−1 bp to −1600 bp) and activated by FC (100 µM) as compared with those transfected with control vector. Deletions from 5′ end of the promoter to −1250 bp, −900 bp, −480 bp and −250 bp (Fig. 4B) yield no luciferase activity, indicating that the promoter −1211 bp to −1600 bp region might be necessary for the promoter activity through NFκB activation. To determine which NFκB binding sites is crucial for the NOS2 gene activation after treated with FC (100 µM), three more constructs (fragment A: −1201 bp to −1280 bp, fragment B: −1211 bp to −1600 bp, and fragment C: −1231 bp to −1600 bp) of NOS2 based on previously predicted two NFκB binding sites on the NOS2 promoter were prepared. As shown in Figure 4 B, luciferase activities of fragment A with one of the functional NFκB binding site (−1224 bp to −1210 bp) was approximately 95% of the full length activity. Fragment B contained the secondary functional NFκB binding site (−1529 bp to −1516 bp) was 1.2 times greater than the full length activity. Fragment C contained both NFκB binding sites evoked 2.6 times activity. Thus, these data suggest that both regions of the NFκB binding sequences are necessary for the maximal NOS2 promoter activity after treated with FC.

### FC Increases NOS2 Expression through ROS Production

The putative effect of ROS on NOS2 expression was studied. To investigate whether antioxidant blocks the FC-induced up-regulation of NOS2, the CEC was pretreated with 5 mM of NAC for 30 min followed by 100 µM FC, and incubated for 24 h. At the end of incubation, cells were harvested for assay. Pretreatment of the cell with NAC prevented the FC-induced increases of transcription of the NOS2 protein (Fig. 5A), NFκB binding onto the NOS2 promoter (Fig. 5B), and the NOS2 promoter activity (Fig. 5C). We also examined whether FC-induced NFκB nuclear translocation in the CEC through ROS-mediated IκBα phosphorylation. As shown in Figure 5D, pretreatment of the CEC with NAC prevented the FC-induced phosphorylation of IκB. In addition, pretreatment of the CEC with Bay 11-7082 (a selective IKK inhibitor) or PDTC (an NFκB inhibitor) abolished the FC-induced increases of the binding of NFκB protein onto the NOS2 promoter (Fig. 5E), suggesting that FC-induced NFκB nuclear translocation was mediated by IκB phospohrylation.

## Discussion

Intracerebral injection of lysed blood, hemoglobin, and FC causes neurodegeneration due to redox cycling of iron complex, increases in hydroxyl radical, lipid peroxidation, oxidative stress, and brain injury [33]. Low dosages of FC (less than 20 nmole) produce dopaminergic toxicity in the midbrain substantial nigra that can be prevented by S-Nitrosoglutathione (GSNO) and NO [34]. In clinical observation, vasospasm is often triggered by lysed blood after SAH, a ruptured brain aneurysm, and bleeding in cerebral spinal fluid of ventricular space. Moreover, 2,2′-dipyridyl, a ferrous iron chelator, prevents delayed vasospasm in a primate model of SAH [15]. Consistently, a review article indicated that ferrous ion might be involved in vasospasm development [35].

In the mouse CEC, FC (0.1 mM) generated ROS, produced little cytotoxicity (unpublished observations), but up-regulated the expression of NOS2 (Figs. 2A, B and C). The induction of NOS2 was triggered by ROS because it was blocked by NAC pretreatment. In previous studies, antioxidants and GSNO/NO prevent the ROS-evoked oxidative stress via the induction of NOS1 expression and related new protein synthesis such as MnSOD, Bcl-2, and thioredoxin [34]. ROS can activate voltage sensitive calcium channel and increase intracellular calcium that could lead to vasospasm [36], [37]; all of which can be blocked by nimodipine, a calcium channel blocker.

NOS2 has been implicated as an important mediator of inflammatory responses during ischemia and reperfusion in rodents [38]–[43]. Rodent NOS2 can be induced by ROS generated by bacterial lipopolysaccharides, interferon-gamma [44], [45] or FC (Fig. 5A). However, the functions of up-regulation of NOS2 in the mouse CEC remain to be studied. The regulation of NOS2 expression is quite complex as it involves a variety mechanisms within a wide range of cell types and species difference. It is known that the human NOS2 promoter is different from that in the mouse by more than 50% [46], suggesting that the functions of NOS2 may have species differences. In AKN1 human liver cells, the induction of NOS2 by cytokine stimulation is mediated by NFκB binding onto the NOS2 promoter ranging from −4,700 bp to −16,000 bp [47]. In the mouse CEC, however, NFκB up-regulated the NOS2 promoter at two functional binding sites near −1500 bp and −1200 bp (Fig. 4). Transfection of the CEC with the construct containing both functional NFκB binding sites (−1211 bp to −1600 bp) induced a significantly higher NOS2 activity as compared with the construct containing only one NFκB binding site (−1201 bp to 1280 bp or −1231 bp to −1600 bp) or a full length of the promoter region (−1 bp to −1600 bp), suggesting that the downstream of the first potential NFκB binding site might contain a binding site for negative regulatory protein. This notion was supported by the result that transfection of the CEC with the construct containing the −1 bp to −1250 bp region, which covered one of the potential NFκB binding site (−1210 bp to −1224 bp), did not induce any significant change of the NOS2 promoter activity. Therefore, NFκB activation of the NOS2 promoter in human is different from that of mouse. To clarify this issue of ROS-activated NFκB for the induction of NOS2, additional future studies need to be performed. The present finding of two functional NFκB binding sites for activation of the mouse NOS2 promoter is in agreement with two proposed NFκB binding sites on the mouse NOS2 promoter based on the Transcriptional search database system (http://www.cbrc.jp/research/db/ TFSEARCH. html).

Nuclear translocation of NFκB (Fig. 3) was mediated by phosphorylation of IκBα, which released NFκB from the complex of IκB-NFκB leading to the translocation of NFκB from the cytoplasm pool to the nucleus and binding onto the promoter for NOS2 transcription. Pre-treatment of the CEC with NAC, a ROS scavenger, prevented the FC-induced increases of IκBα phospohrylation (Fig. 5D), NFκB binding onto the NOS2 promoter (Fig. 5E), the NOS2 promoter activity (Fig. 5C), and the level of NOS2 protein (Fig. 4A). Taken together, these data suggest that an increase of ROS might contribute to FC-induced up-regulation of NOS2 through activation and binding of NFκB onto the NOS2 promoter of the mouse CEC. These results are consistent with early reports [44], [48]. However, the issue of how IKK is activated after FC treatment has not been addressed in this study. A previous study on cultured vascular smooth muscle cells has suggested that statins diminish NFκB activation elicited by oxidative stress through the inhibition of IKK-1/-2, p38 mitogen-activated protein kinase (MAPK), and p42/44 MAPK activation [49]. The molecular mechanism underlying ROS-induced IKK activation in the FC-treated CEC is still not clear and deserves further investigation. Since NOS2 could play central roles in inflammation mediated by microglia and macrophages in the central nervous system, it will be interested to know whether the similar pathways are involved in NOS2 induction in other inflammatory cells after ischemia or FC treatment. Although increases of interferon-γ-inducible macrophage nitric oxide generation through the NFκB-dependent pathway [50] and involvement of ROS in activation of NFκB in neutrophils [51] have been demonstrated, the direct evidence for molecular signaling pathways involved in ischemia- or FC-induced NOS2 induction in microglia or macrophages has not been found. Whether this signaling pathway involved in the FC-induced NOS2 induction is unique to cerebral endothelial cells still needs further investigation. In conclusion, this study provides evidence that ROS produced by FC small molecular weight iron complex caused no apparent cytotoxicity in the mouse CEC, while activated phosphorylation of IκB and nuclear translocation of NFκB resulted in activation of the NOS2 promoter and related protein transcription (Fig. 6). The most interesting findings are the identification of two functional NFκB binding sites for the activation of the mouse NOS2 promoter.
